# Good Outcome for an Individual with Severe Facial Anomalies and Hypogonadotropic Hypogonadism: A Consequence of His Cognitive Function, Pragmatic Approach, and Temperament

**DOI:** 10.1155/2021/9957218

**Published:** 2021-06-12

**Authors:** Timothy L. Groh, William Zarella, Peter A. Lee

**Affiliations:** ^1^Penn State College of Medicine, Hershey, PA, USA; ^2^Cleo Hogan Law Firm, Clarksville, TN, USA; ^3^Department of Pediatrics, Penn State College of Medicine, Hershey, PA, USA

## Abstract

The multiple factors that determine outcomes for individuals with visible developmental errors and/or atypical development of the reproductive system are not fully understood. This case report of an individual with Bosma arhinia microphthalmia syndrome causing severe facial anomalies and hypogonadotropic hypogonadism is used to highlight factors that impacted his adjustment from childhood through adulthood. Key factors include his temperament, intact cognitive ability, and pragmatic approach for controlling his physical and social environment. His successful adjustment even in the face of significant early life challenges demonstrates that positive outcomes are attainable for individuals with significant developmental errors. His story and experiences with the health-care system offer insight into some factors that may be pertinent to resilience and lifelong adjustment for patients with similar conditions and the importance of continually seeking the patient's perspective to tailor treatment across the lifespan.

## 1. Introduction

Individuals who have significant developmental errors involving parts of the body that are visible and/or atypical development of the reproductive system are at risk for maladjustment, poor body image, and other psychological issues impacting their quality of life (QoL). The multiple factors that determine outcome among those with such defects are poorly understood, and existing research has significant methodological challenges [1–12]. To better understand these factors, here, we present a case report of a man who was born with both a visible facial condition and incomplete development of the reproductive system, had a “miserable and sad” (his terms) childhood and adolescence, but has a well-adjusted QoL as an adult. The excellent QoL for this man, now in his 40s, is based on his relationships with others (spouse, children, and friends), employment, home, and financial and general social status.

Additionally, several points regarding his experiences in the health-care system are noted so that providers can better understand his perspective and improve care for other patients who face similar challenges. It is clear that his outcome is a consequence of his disposition, his cognitive ability, and his rational approach and choices for his activities and interactions with others. A pragmatic decision he made as a young man was crucial and dramatically changed, for the better, the course of his life. He provided written informed consent for publication of his case history.

## 2. Case Presentation

### 2.1. Infancy and Childhood

This man was born with what was diagnosed at the time as Binder syndrome, involving severe hypoplasia of his nose and midface [13]. His diagnosis was updated to Bosma arhinia microphthalmia syndrome (BAMS) following genetic testing. Current evidence suggests that BAMS involves pathogenic variants in the protein-coding gene Structural Maintenance of Chromosomes Flexible Hinge Domain Containing 1 (SMCHD1) [14, 15], specifically in the extended ATPase domain [16]. The resultant arhinia is associated with hypogonadotropic hypogonadism, likely secondary to defective GnRH neuron production in, or migration from, the olfactory placode [17, 18].

Childhood for this man was “horrific” because of the midfacial defect with a cleft that extended from his maxilla to between his eyes. As a consequence of this and the stress that resulted within his family, frequently precipitated by responses and reactions of others and exacerbated by his mother's angry reaction to negative comments, he describes his childhood as “sad and lonely,” saying that he was “abandoned, fostered, rejected, and neglected.” He had multiple surgical procedures consistent with the state of the art for the time. The consequences of his nuclear family's reactions, including the separation of his parents, negatively impacted his care and involved his mother punishing him by withholding food and locking him in his bedroom. He was placed in foster homes more than twelve times from age 2 years to 18 years. At age 11, he went to live with his father where he stayed until age 13 when his father died of cancer.

### 2.2. Teenage Years

After the death of his father, he remained in foster care until he was 18 when he lived independently and worked to support himself. In retrospect, he observes that while he may have seemed outwardly to be accepting his plight well; inwardly, he was “crushed, disappointed, and sad.” Nevertheless, he now observes that his predicament forced him to grow up to cope with these issues as an adult. He comments that “self-raising does that” and elaborates that by having to frequently fend for his own basic needs such as food, water, and safety, he developed a “survival mode” that has enabled him to face difficulties directly.

His hypogonadotropic hypogonadism was suspected when he was referred for lack of pubertal development at age 13 years, and at that time, he was told that he would be unable to father children. Physical examination showed a prepubertal penis and small, unusually firm testes within the scrotum. He was treated with depo testosterone injections to stimulate pubertal development with dosage increases over about three years when he was on a full adult replacement dosage. He was told that he had hypogonadotropic hypogonadism, with his medical records indicating a diagnosis of Kallmann syndrome. He recalls that he was not familiar with this term until he obtained a copy of his medical records at age 22.

Subsequently, he was treated with human chorionic gonadotropin (hCG) followed by a combination with human menopausal gonadotropin (hMG) with intermittent examination of semen. No mature or immature sperm were ever seen. This was followed by a period of pulsatile GnRH therapy via an infusion pump, also without sperm production. Testicular biopsy at age 18 showed immature sperm cells.

As a teenager, he had many negative thoughts about group homes, foster care, multiple surgeries, and lack of sperm production. He retrospectively says that he ended up within his “own world” since he did not “look normal” and had failure of both testosterone and sperm production. There was a particularly negative incident when a foster mother falsely accused him of sexual abuse of her three-year-old daughter. This was based upon the mother's report that the daughter said “(he) taught me to peepee.” He recalls being left by the foster parents to supervise their other children, so there were times where he had to tell the other children to use the bathroom. He feels that this false, unfounded accusation was the most damaging event to him. Consequently, he learned to be very guarded about things he said to and about people while keeping a physical distance. However, he had no choice but to remain in the foster system until he was 18 years old when he, on his own, found a place to live and began to work. He has always been employed since.

### 2.3. Adulthood

He decided as a young adult that he needed to share and confess the multiple issues that had been problematic for him. He did this confidentially with a friend and, as a consequence of their discussion, he decided that he needed to move away from the area where people knew him and his situation. He decided that he wanted “a fresh start” because people where he lived and grew up knew of the “terrible” events of his childhood. On a regular basis, people who knew of the “bad things” from his past made comments. Even though these comments were often regarding how well he had performed in spite of his past, these were all too frequent unwelcome reminders and made him feel that everyone expected him to turn out poorly. He has never heard such comments about his past since he moved more than 20 years ago; however, he is still subjected to daily comments about his facial appearance and these continue to bother him “a great deal.”

Since the company where he worked had offices in multiple locations, he applied for work and was able to transfer his employment with the same firm. This involved moving to another state more than 550 miles away. When he told a female friend about his move, she said she would move with him. This was the same friend in whom he had initially confided all the details and issues of his past. She was a single parent with two children. Previously, she had introduced him to her parents, and they told her not to have anything to do with him apparently because of his facial appearance and his past. She later became his wife providing him with two stepchildren and remains his wife more than 20 years later.

He indicates that his adult life has been great, but only after moving. His wife is a Christian while he is not religious. As a child, he was bothered about talk of predestination and free will and had considerable guilt, asking “why God would let this happen.” His current perspective is that by “letting this go,” it helped him. Another perspective that gives him purpose is a belief that everyone's individual story is special and that by sharing his own story he can help other people. Currently, he is “not bothered” by what those around him believe either religiously or politically.

He indicates that it is easy for him to make adult friends, and as a result, he has had many male and female friends of all ages during his adult life. However, he did comment that he is not good at retention of friendships. He attributes this to the fact that he tends to overreact to situations and has “no filter” which leads him to avoid “sugarcoating” things other people may not want to hear. He observes that he feels that he is like his mother in this regard. His earliest memories of her are of her overreacting to people who made comments about him and his appearance, not uncommonly resulting in physical fighting. Interestingly, although he moved from the area where he grew up, he has maintained contact with some friends and family members, including his mother, and visits them occasionally, especially at holiday times. He comments that he puts in considerable effort to maintain relationships, even if that effort is not always reciprocated, and he tends to form strong attachments. He has had counseling throughout his adult life from age 19 until a couple years ago, and he thinks it has helped him as an adult by making him more self-aware.

For the past ten years, he has worked for a law firm. His tasks involve aiding people with disabilities in obtaining finances and other support. He feels that the skills and empathy that have made him successful in this career are a natural extension from his own life experiences and constantly facing unfavorable social expectations. Notably, he says that he does not view himself as sick but instead sees himself as very fortunate, adaptable, and capable of doing anything if he sets his mind to it.

He has been invited to talk to foster support groups about being a foster-kid, including back in the area where he grew up. He has found this to be a positive experience. He often thinks about being a motivational speaker because he has found that people are fascinated about how he could “turn out” after a childhood of “all challenging circumstances.”

He now says, concerning his appearance, that he cannot imagine being any other way. In fact, he attributes some of his “drive to survive” to his condition, noting that, from the moment he was born, he had to learn to breathe exclusively through his mouth in order to live. When he was recently told he had a genetically verified diagnosis of BAMS at 41 years of age, he learned that the facial midline defect sometimes involves eye developmental problems and blindness. Even though he was born with the absence of a nose, his reaction was thankfulness that except for a missing lacrimal duct, his eyes and vision are normal.

### 2.4. Health-Care Experiences

Though many of the challenges he faced while growing up were beyond the reasonable scope of medical care, interactions with the health-care system and health-care workers are important touchpoints for offering support and uncovering how these external challenges may be impacting outcomes. While acknowledging that each individual's experience may be different, his story may offer insight to help better serve other patients. Beginning with some of his earliest experiences with the health-care system, he says that his mother would often use the hospital as the “babysitter.” He remembers being left alone after his surgeries and calling his mother on the phone to beg her to come and visit. He remembers being comforted by nurses and doctors when he was left alone, and he says that these experiences taught him empathy, a skill that he now sees as very important in his adult life. When, at age 13, he was told that he would be unable to father children, he remembers feeling “that piece of the puzzle shattered” because to that point, an important hope for him was to one day have his own family. He says that this disclosure was delivered very bluntly, and to this day, he can recall exactly how it made him feel and even fine details such as what clothes he was wearing at the time. While full disclosure is the goal and this news may be traumatic at different stages of development, he expresses the wish that this piece of information had been delivered later on when he was an adult. He is also supportive of full disclosure and has an intense desire for knowledge about his medical conditions and care, so he appreciates when providers offer him detailed explanations of diagnoses. As he continued to have frequent interactions with the health-care system during his teenage years, he says that he got used to “going along with what doctors said” and that iterative surgeries and appointments became the routine that shaped his “own world.” He says that he never felt pressured to assent to any treatment that he did not want, but eventually, he decided that he did not want any more surgeries after age 16. During these teenage years, he also says that he was not always forthcoming and fully honest with providers because he felt “confused and embarrassed” about aspects of his condition. Nevertheless, he was still able to form trusting relationships with caring providers and has maintained contact with a few providers into adulthood. He cautions that other patients who face multiple medical and psychosocial challenges may seem externally well adjusted, but it is possible that internally they are struggling to cope and adjust, similarly to what he went through. Lastly, as a participant in biomedical research throughout his adult life, he expresses a desire for regular communication and updates from researchers so that he does not feel like “just a number.”

## 3. Discussion

This case of successful adjustment and excellent QoL in adulthood for this man born with BAMS resulting in hypoplasia of his nose and midface and hypogonadotropic hypogonadism demonstrates that positive outcomes are attainable for individuals with significant visible developmental errors and/or atypical development of the reproductive system, in spite of substantial early-life difficulties. His life history provides a rich exploration of resilience, a multidimensional construct encompassing the dynamic interaction between individual traits and external resources that combine to produce a more positive outcome than expected in the face of adversity [19, 20]. Resilience has been of recent interest as it relates to psychological coping and recovery from medical illness as well as risk versus protection for psychiatric illness [20, 21]. Components of resilience may be amenable to intervention in order to increase the likelihood of more positive future outcomes [20, 22]. For example, the characteristic intact cognitive ability of patients with BAMS [23] can be leveraged to bolster academic investment and help them secure meaningful work like this man was able to achieve. In combination with this strength-based approach, simultaneous efforts such as integrated medical and behavioral health care may help address any hindrances to resilience. For example, this man feels that he has benefited from counseling, but for him, it has been something he has mostly had to seek out in adulthood. Notably, resilience may vary even within a single individual across circumstances and across the lifespan, adding to the variability in developmental trajectories between individuals. The division of this man's story into periods of childhood, teenage years, and adulthood provides freeze frames of evolving traits, fluctuating environmental circumstances, and increasing capacity to shape those circumstances in relation to a life course perspective on his adjustment and QoL.

For this man, his temperament, cognitive ability, and pragmatic approach have been key moderators of his well-adjusted adulthood. His early adulthood decision and ability to move and change his environment was also crucial in supporting his current QoL. His story and experiences with the health-care system offer insight into some factors that may be pertinent to resilience and lifelong adjustment for patients with similar conditions as well as the vital importance of continually seeking the patient's perspective to best tailor care to their unique circumstances and desires across the lifespan.

## 4. Key Points

Individuals with visible developmental conditions and/or atypical development of the reproductive system are at risk for maladjustment, though the specific factors impacting their QoL are not fully understood. Health-care professionals are adult models for pediatric patients, especially those who frequently interact with the health-care system. They may provide the hidden curriculum for basic prosocial behavior such as empathy that some children do not otherwise see frequently modeled. Full disclosure of medical conditions should proceed in a developmentally appropriate fashion via a team-based approach. We practice closed-loop communication with patients involved in research in order to share discoveries and respect their altruistic contributions to medicine. An individual patient's resilience may fluctuate across different circumstances and across the lifespan. The index of suspicion for maladjustment should also shift with context so that timely and appropriate referrals can be made for counseling and peer-to-peer support.

## Data Availability

The individual medical records used to support this study have not been made available to protect patient privacy.
